# Satisfaction with Life of Schizophrenia Outpatients and Their Caregivers: Differences between Patients with and without Self-Reported Sleep Complaints

**DOI:** 10.1155/2013/502172

**Published:** 2013-10-27

**Authors:** Sofia Brissos, Pedro Afonso, Fernando Cañas, Julio Bobes, Ivan Bernardo Fernandez, Carlos Guzman

**Affiliations:** ^1^Centro Hospitalar Psiquiátrico De Lisboa (CHPL), 1170 Lisbon, Portugal; ^2^Janssen-Cilag Farmacêutica Ltd., Estrada Consiglieri Pedroso 69-A, Queluz de Baixo, 2734-503 Barcarena, Portugal; ^3^Departamento de Psiquiatría, Universidad de Oviedo, Centro de Investigación Biomédica en Red de Salud Mental (CIBERSAM), Asturias, 33006 Oviedo, Spain; ^4^Hospital Público Dr. R. Lafora, 28031 Madrid, Spain; ^5^Janssen Pharmaceutical, 28042 Madrid, Spain

## Abstract

Patients with schizophrenia often present sleep complaints, but its relationship with general satisfaction with life (SWL) and burden for caregivers has been understudied. We aimed to assess the differences in SWL between patients with and without self-reported sleep disturbances and that of their caregivers. In a noninterventional study, 811 schizophrenia adult outpatients were screened for their subjective perception of having (or not) sleep disturbances and evaluated with the Brief Psychiatric Rating Scale (BPRS) and the Pittsburgh Sleep Quality Index (PSQI). Patients self-reporting sleep disturbances were significantly more symptomatic (*P* < 0.001), presented significantly worse family support (*P* = 0.0236), and self-reported worse SWL in all domains. Caregivers of patients with schizophrenia self-reporting sleep disturbances also reported worse SWL in all domains, as compared to caregivers of patients without subjective sleep disturbances. Patient and caregivers' SWL was significantly correlated to patients' quality of sleep (*P* < 0.0001 for all domains). Patient' and caregivers' SWL was negatively affected by patients' poor quality of sleep. We found that patients self-reporting sleep disturbances showed greater symptom severity, worse quality of sleep, worse SWL, and less caregiver support. SWL was also worse for caregivers of patients with schizophrenia reporting sleep disturbances.

Patients with schizophrenia often present sleep complaints [1], even while being medicated and clinically stable, which can negatively affect their quality of life [2] and be sufficiently severe to warrant clinical attention.

Nowadays, besides symptomatic control, the aim of clinicians is to improve schizophrenia patients' social functioning, quality of life, and satisfaction with life (SWL). To accomplish this, physiologic sleep may be necessary. 

General SWL has been associated with symptoms, cognition, health-related quality of life, and medical comorbidity [3–5]. Higher burden has been reported for caregivers of schizophrenia patients with higher symptom severity, disruptive or difficult behavior, younger age, and patients' need for care [6–10]. 

Given the importance of sleep in schizophrenia, we aimed to assess the differences in SWL between patients with and without self-reported sleep disturbances and that of their caregivers, as well as the degree of family support, since we found no previous reports on this subject.

In a multicenter, Iberian, cross-sectional, noninterventional study, 811 outpatients with a diagnosis of schizophrenia, aged ≥18 years, and with no changes in antipsychotic treatment for at least 6 months were screened for their subjective perception of having (or not) sleep disturbances [11]. We excluded patients with sleep disturbances related to disorders other than schizophrenia (e.g., nightmares, nocturnal fears, and restless leg syndrome), schizoaffective disorder, organic impairment, or cognitive deficits. 

Patients were evaluated with the Brief Psychiatric Rating Scale (BPRS) and the Pittsburgh Sleep Quality Index (PSQI). Patient and caregiver satisfaction with several domains of life (i.e., job, family, sexual and social life, health, and general satisfaction) was evaluated with a visual analogue scale. Family support degree was rated by the investigator as (1) very high, (2) high, (3) medium, (4) low, and (5) null. We compared the two groups using parametric Student *t*- or Snedecor *F*-tests. The association between quality of sleep and patients' and caregivers' SWL was calculated with Pearson's correlation coefficient.

Of the 811 patients, 401 self-reported having sleep disorders and 410 denied them. Patients were predominantly male (66%), 84% lived accompanied, and the majority was professionally inactive (76%). There were no statistically significant differences between the groups regarding age, gender, educational level, employment status, age of diagnosis, illness duration, and type of antipsychotic treatment.

Patients self-reporting sleep disturbances were significantly more symptomatic (mean (SD) BPRS 12.8 (8.27) versus 16.6 (9.43), *P* < 0.001). 

Patients self-reporting sleep disturbances presented significantly worse family support (Chi^2^ = 2.2636, *P* = 0.0236) and self-reported worse SWL in all domains (Table 1). Caregivers of patients with schizophrenia self-reporting sleep disturbances also reported worse SWL in all domains, as compared to caregivers of patients without subjective sleep disturbances (Table 1).

Patients' and caregivers' SWL was significantly correlated to patients' quality of sleep (measured by the PSQI) (*P* < 0.0001 for all domains).

In this Iberian population, patients' and caregivers' SWL was negatively affected by patients' poor quality of sleep. Importantly, we studied patients' perceptions, which may in themselves be biased; therefore, objective evaluation of sleep (i.e., polysomnography or actigraphy) is needed in future studies. The inclusion of patients taking benzodiazepines and other psychotropics may have also biased the results, and stimulant (e.g., coffee, tea, etc.) and alcohol use and weight were not controlled for. Furthermore, patient and caregiver satisfaction may be influenced by differences in mental health provision, social network, and other cultural factors [12], which were not evaluated in our study. 

Sleep disturbances may reinforce altered sleep patterns, cognitive deficits, and social engagement associated with schizophrenia, possibly with a negative impact on both patients' and caregivers' SWL. For these reasons, treatment plans for schizophrenic patients should explicitly incorporate strategies to deal assertively with complaints of poor sleep quality.

Limitations include the lack of objective evaluation of sleep (i.e., polysomnography or actigraphy) and of control for medication, stimulant (e.g., coffee and tea), and alcohol use. Moreover, we cannot be sure if the two groups are comparable on unobserved baseline variables, and the subanalysis was not powered.

In our Iberian study of 811 outpatients with schizophrenia we found that patients self-reporting sleep disturbances showed greater symptom severity, worse quality of sleep, worse SWL, and less caregiver support. SWL was also worse for caregivers of patients with schizophrenia reporting sleep disturbances. A growing body of research has revealed that improvements in several factors (i.e., adherence, cognition, symptoms, and access to services) may be necessary to improve patients' SWL [3]. Family support and sleep hygiene may also have beneficial effects on these patients' SWL. Future longitudinal studies should explore if insomnia is only a characteristic symptom of schizophrenia or whether ongoing sleep disturbances might also affect the course and the prognosis of the illness.

## Figures and Tables

**Table 1:** Patients' and caregivers' level of satisfaction with life (SWL).

	Patients without sleep disturbances (*N* = 410)	Patients with sleep disturbances (*N* = 401)	Student t-test	*P* value
	Mean (SD)	Mean (SD)		
Patient satisfaction				
Job satisfaction	4.3 (2.84)	3.4 (2.73)	4.36	<0.0001
Family satisfaction	6.6 (2.34)	5.8 (2.56)	4.86	<0.0001
Sexual satisfaction	3.5 (2.83)	3.0 (2.70)	2.57	0.0105
Social life satisfaction	5.4 (2.28)	4.4 (2.35)	5.97	<0.0001
General satisfaction	6.0 (1.91)	5.0 (2.08)	6.99	<0.0001
Satisfaction with health	6.5 (1.88)	5.6 (2.11)	6.65	<0.0001

	(*N* = 212)	(*N* = 222)		

Caregiver satisfaction				
Job satisfaction	4.1 (2.94)	3.3 (2.57)	2.94	0.0035
Family satisfaction	6.4 (2.33)	5.4 (2.37)	4.43	<0.0001
Sexual satisfaction	3.9 (2.72)	3.1 (2.65)	3.23	0.0013
Social life satisfaction	4.8 (2.10)	3.8 (2.18)	4.75	<0.0001
General satisfaction	5.8 (1.88)	4.7 (1.91)	5.85	<0.0001
Satisfaction with health	6.5 (1.82)	5.5 (2.07)	5.35	<0.0001
